# Dataset of working mPEG-alkyne with scCO_2_

**DOI:** 10.1016/j.dib.2021.106907

**Published:** 2021-02-25

**Authors:** S. López, M.J. Ramos, J.M. García-Vargas, M.T. García, J.F. Rodríguez, I. Gracia

**Affiliations:** Department of Chemical Engineering, Institute of Chemical and Environmental Technology (ITQUIMA), University of Castilla-La Mancha, Avda. Camilo José Cela 12, 13071 Ciudad Real, Spain

**Keywords:** ScCO_2_, MPEG-alkyne, Sorption, Mathematical models

## Abstract

This article contains data related to the research article entitled “Carbon dioxide sorption and melting behavior of mPEG-alkyne”. The presented data gives information on the thermodynamics properties of the solvent and the polymer. The time saturation of mPEG-alkyne in supercritical carbon dioxide (scCO_2_) was evaluated in a high-pressure variable volume cell in different period of time at different pressure at the same temperature.

The effects of pressure and temperature on the density of CO_2_ when it is above supercritical conditions are determined with Sanchez Lacombe and Bender Equation and compared with the NIST database and values of equation of Bender. The characteristic parameters of CO_2_ were determined with the equations proposed by Chengyong Wang et al. [1] and the sum of squared error was calculated for each parameter.

Furthermore in this work the solubility data of scCO_2_/polymer mixture were correlated with Sanchez Lacombe Equation of State (SL EOS) and Heuristic model proposed by Irene Pasquali et al. [2]. This work describes the methodology for solving the SL EOS between the polymer and scCO_2_ and the procedure of determining the solubility parameter with the group contribution method necessary to apply the heuristic model is described.

## Specifications Table

**Table:** 

Subject	Chemical Engineering
Specific subject area	Thermodynamics
Type of data	Tables, figures
How data were acquired	High-pressure variable volume cell (Cell model was ProVis 500 from Eurotechnica), the solver system used the nonlinear programming algorithm generalized reduced gradient (GRG) and data published in literature.
Data format	Raw and Analysed
Parameters for data collection	The saturation time in mPEG-alkyne was measured at 313 K and 11 MPa. The characteristic parameters for CO_2_ were compared and determined at temperatures and pressures in the range of interest to determine CO_2_ density was at 308 K and 318 K following SL EOS model. The Small method was used to calculate the solubility parameter, δ. This parameter was necessary to apply the heuristic model in our experimental data. The sum of squared error was calculated with the different mathematical models applied in the experimental data.
Description of data collection	Saturation time was estimated with high pressure cell variable volume. The characteristic parameters are available in literature.
Data source location	University of Castilla-La Mancha, Ciudad Real (Spain)
Data accessibility	Data are available in this article
Related research article	https://doi.org/10.1016/j.supflu.2021.105182.

## Value of the Data

• The experimental sorption data are required to characterize systems that will be used in loading and release of drugs with medical applications.
• The data were correlated by different mathematical models of equations of state to obtain adjustment parameters. These parameters will permit to predict sorption data in conditions different to those used in our experiments.
• The reader can use our data to evaluate the influence of pressure and temperature on the sorption of CO_2_ in mPEG-alkyne.
• The study of melting point provides information on the pressure required to melt the polymer and to produce a saturated liquid solution, which is a parameter of major importance, especially for the preparation of a conjugated drug-polymer in scCO_2_, with medical interest.
• The sum of squared errors (SSE) data indicate that the calculation method applies to other systems of interest for the pharmaceutical, food and chemical industry mainly.

## 1 Data Description

**Fig. 1:**
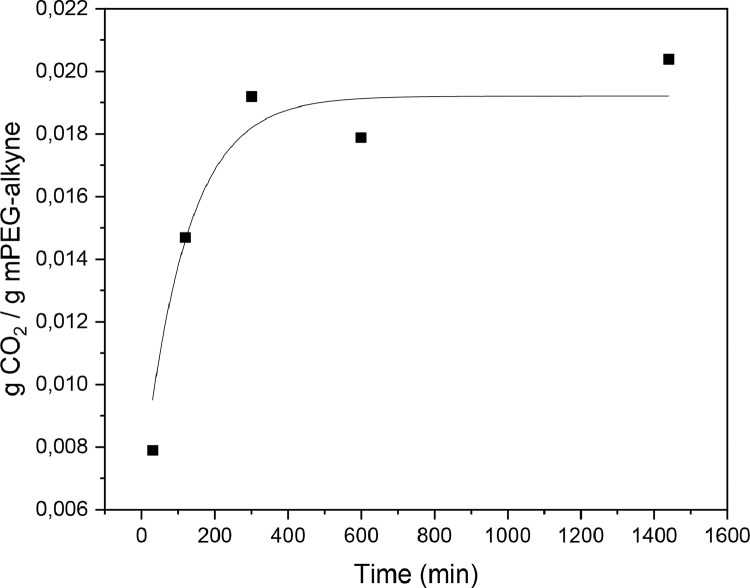
Sorption of CO_2_ in mPEG-alkyne at 313 K, 11 MPa at different times.

The data presented in this article include the experimental data used to determine the saturation time between mPEG-alkyne and scCO_2_ at 313 K and 11 MPa, Fig. 1.

Saturation time can be defined as the time required in order to reach the equilibrium state between the polymer and supercritical carbon dioxide. Taking into account, the operating conditions, we measured the saturation time of mPEG-alkyne at 313 K and 11 MPa. It can be observed, in Fig. 1 that the measured saturation time was 300 min, as the CO_2_ solubility from than point remained practically constant.

Table 1 contains the data set of characteristic parameters used to determine CO_2_ density at 308 K and 318 K following Sanchez-Lacombe EOS and the values were compared with the density values recorded in the NIST database [3].

**Table 1:** Sanchez-Lacombe characteristic parameters for CO_2_, the SSE_Bender_ and SSE_SL_ compared with NIST data [3,4].

								308 K	318 K
Set	P* (MPa)	T* (K)	ρ* (g/cm^3^)	P_c_^a^ (MPa)	T_c_^a^ (K)	ρ_c_^a^ (g/cm^3^)	Ref.	SSE^b^_SL_ (%)	SSE^b^_SL_ (%)
**1**	412.6	316.1	1.369	8.745	302.66	0.4216	[5]	1.495	0.988
**2**	574.5	305	1.51	9.087	316.10	0.4230	[6]	1.021	0.096
**3**	438.8	314.8	1.416	8.990	304.44	0.4313	[7]	1.370	0.848
**4**	427.7	338.7	1.4055	9.660	318.42	0.4419	[8]	0.975	0.086
**5**	369.1	341.2	1.253	8.696	316.75	0.3993	[9]	1.029	0.294

a Critical properties of CO_2_ are T_c_=304.3 K, P_c_= 7.38 MPa and ρ_c_=0.471 g/ml.

b $\text{SSE}=\sum_{i=1}^{n}\left(x_{i}^{\text{exp}\;\text{data}}-x_{i}^{\text{calc}\;\text{data}}\right).$

**Fig. 2:**
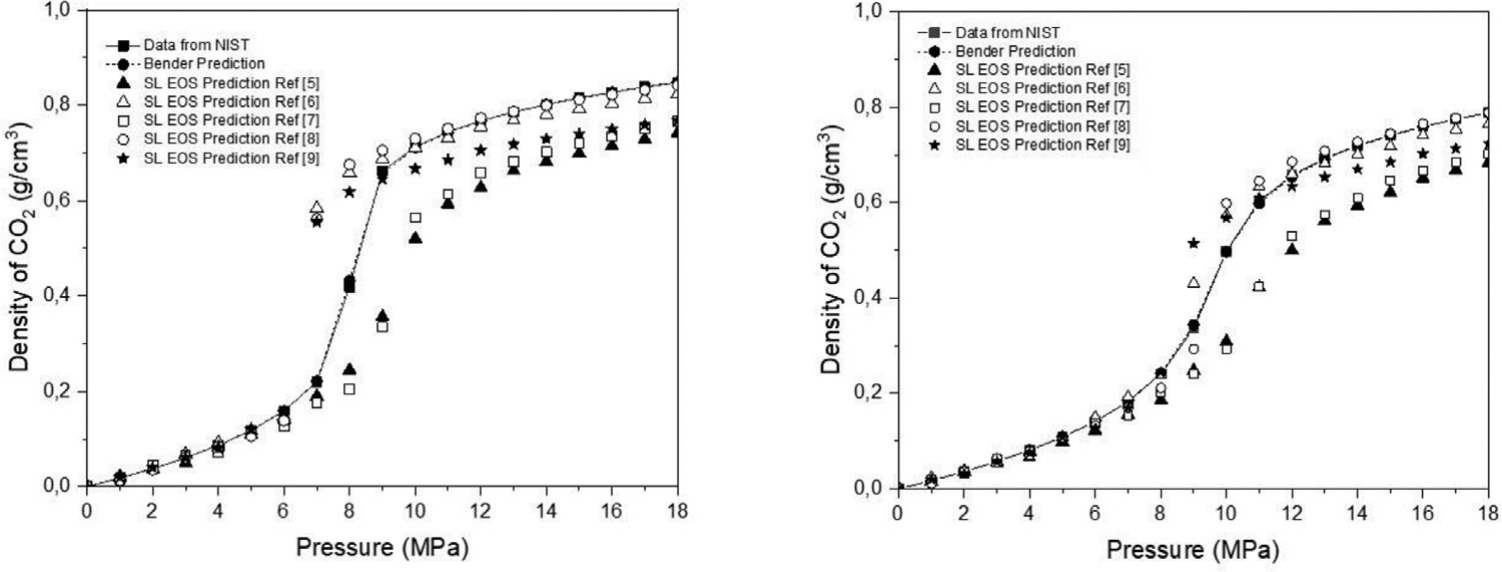
Comparison of Bender equation and Sanchez-Lacombe prediction of CO_2_ density at 308 K (left figure) and 318 K (right figure) with the density reported in NIST. The different characteristic parameters used are referenced in the legend of the graph.

Additionally, the CO_2_ density determined with the equation of Bender was compared to the density reported in the NIST data base and finally was calculated the sum of squared errors (SSE). The SSE of Bender data and NIST was 0.00103% for 308 K and 0.004% for 318 K. In Fig. 2, the comparison of CO_2_ density between the Bender equation and Sanchez-Lacombe with respect to the density reported in NIST can be observed.

The model prediction was fairly good at low pressure, however, as pressure was increased, the deviation of the model from the NIST data increased. Therefore, characteristic parameters not necessarily provide an accurate prediction of the CO_2_ density. For instance, sets 3 and 4 accurately predicted the critical temperature of CO_2_, but the smallest SSE was obtained when using the set 4. Therefore, the characteristic parameter set was chosen in order to obtain the lowest SSE in the prediction of critical properties of CO_2_.

Table 2 contains the solubility parameter with the group's contribution method. Atomic group contribution methods have been used to estimate the solubility parameter. The sets of group Small, Hoy and van Krevelen seem to be most comprehensive. Small's values were derived from measurements of the heat of vaporization. Hoy's values were derived from vapor pressure measurements. The group contribution values of van Krevelen are based on cohesive energy data of polymer. The contribution of Small, van Krevelen and Hoy for mPEG-alkyne are summarized in Table 2.

**Table 2:** Group contributions to solubility parameter.

	F (MPa^0.5^ cm^3^ mol^−1^)			
Group	Small	van Krevelen	Hoy	Ref.
$-CH_{3}$	437	420	303	[10]
HC≡C−	583	–	–	[10]
$-CH_{2}$	272	280	269	[11]
−O−	143	255	235	[11]

The density of mPEG-alkyne at 298,15 K is 1.127 g/ml. The solubility parameter of mPEG-alkyne was calculated with the Small method of group contribution as 17.6082 MPa^1/2^ or 8.8041 cal^1/2^ cm^−3/2^. The density values and solubility parameters at different temperature, pressure and mole fraction of mPEG-alkyne are shown in Table 3.

**Table 3:** Density values and solubility parameters at different temperatures and pressures and mole fraction of mPEG alkyne at 308 K and 318 K at different pressures.

Temperature	Pressure	Density, ρ_a_	δ
K	MPa	g/cm^3^	(cal/cm^3^)^1/2^
308	8	0.4192	3.6709
308	9	0.6621	5.7987
308	10	0.7128	6.2425
308	11	0.7440	6.5151
308	13	0.7857	6.8807
308	15	0.8151	7.1378
308	17	0.8381	7.3395
318	8	0.2411	2.1111
318	9	0.3375	2.9559
318	10	0.4983	4.3637
318	11	0.6032	5.2822
318	13	0.6937	6.0747
318	15	0.7420	6.4978
318	17	0.7755	6.7918

The data set of square errors of experimental solubility with the solubility calculated with the mathematical models of Henry, Dual mode, Sanchez Lacombe and the heuristic model is shown in Table 4.

**Table 4:** SSE between experimental and mathematical models.

Pressure	308 K			
MPa	SSE_Henry Model_	SSE_Dual Mode_	SSE_SL EOS_	SSE_Heuristic model_
80	0,01,168,103	0,00,012,936	0,00,113,602	0,00,000,082
90	0,01,050,369	0,00,005,365	0,00,000,582	0,00,013,461
100	0,00,968,541	0,00,000,647	0,00,000,233	0,00,018,339
110	0,00,908,498	0,00,000,952	0,00,001,210	0,00,194,408
130	0,00,826,474	0,00,028,293	0,00,025,853	0,00,068,548
150	0,00,773,183	0,00,112,027	0,00,083,015	0,00,164,968
170	0,00,735,834	0,00,276,055	0,00,165,991	0,00,006,680
**Total**	0,06,431,002	0,00,436,276	0,00,390,486	0,00,466,486


Pressure	318 K			
MPa	SSE_Henry Model_	SSE_Dual Mode_	SSE_SL EOS_	SSE_Heuristic model_
80	0,0256	0,000,753,467	0,000,177,938	1,33753E-05
90	0,026,860,428	0,000,699,096	0,000,281,966	6,42469E-05
100	0,02,476,788	0,000,181,041	4,29248E-08	9,39312E-05
110	0,023,232,456	5,71112E-07	9,89037E-05	0,000,473,956
130	0,021,134,897	0,000,612,832	1,55234E-05	0,000,150,223
150	0,019,772,125	0,002,602,863	8,52215E-05	9,87041E-05
170	0,018,817,015	0,006,118,898	0,000,541,561	4,50077E-08
**Total**	0,000,894,481	0,001,201,157	0,010,968,768	0,160,184,801

## 2 Experimental Design, Materials and Methods

The saturation time of mPEG-alkyne/CO_2_ was determined by employing a high pressure cell of variable volume. The sorption data were estimated experimentally taking as reference the saturation time and subsequently the sorption data were correlated with several mathematical models, as Henry model, Dual-Mode Sorption, SL EOS and Heuristic model.

### 2.1 Evaluating the saturation time of mPEG-alkyne/CO_2_ system

**Fig. 3:**
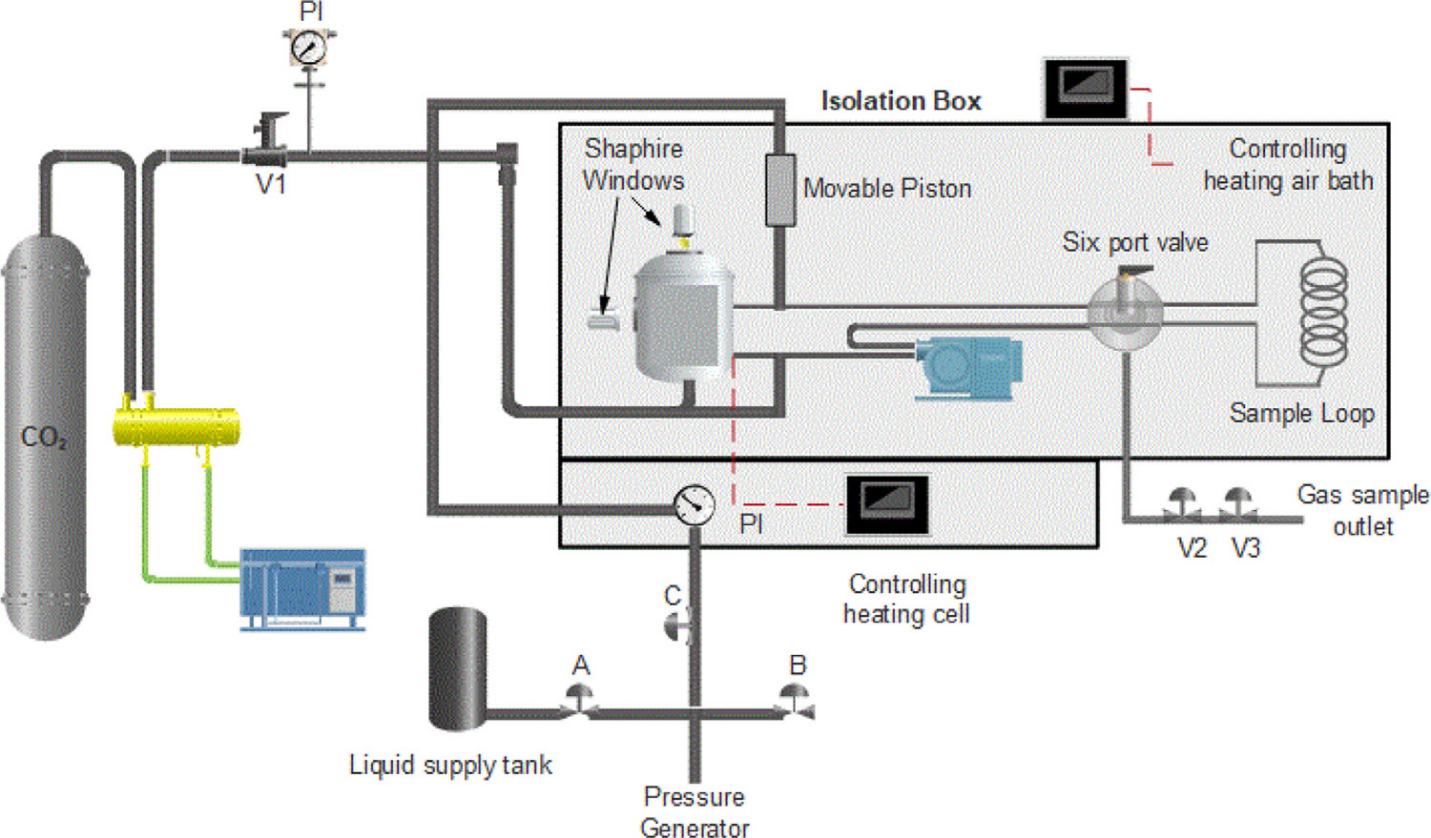
Schematic diagram of the variable high-pressure view cell for sorption measurements.

The saturation time was measured with high pressure cell of variable volume (Fig. 3).

In this study, the sorption measurement was carried out with ex situ gravimetric method, in which the sample is saturated of CO_2_ until the equilibrium condition. The time for reaching phase equilibrium was determined by several preliminary experiments, in which samples were taken after 30, 120, 300, 600, 1440 min. After allowing the polymer to saturate with CO_2_ the cell was depressurized [12].

The weight gain of the mPEG-alkyne due to sorption of CO_2_, was obtained after 300 min. The equilibrium solubility was calculated as mass of CO_2_ absorbed per gram of mPEG-alkyne, as indicated the Eq. (1).

From Eq. (2) and knowing w_o_, weight of polymer before pressurization process, the weight of mPEG-alkyne was determined. The CO_2_ mass fraction was determined with Eq. (3) and ρ_CO2_ was determined with the Equation of Bender [13], with SL EOS and with the NIST database [3].

These data are checked by volumetric measurements, which consist on the saturation of polymer in a previously calibrated crucible. When the equilibrium is reached, the crucible is vented, and the volume of CO_2_ is measured through a turbine flowmeter.

(1) $$S\;\left(\text{wt}.\;\text{fraction}\right)=\frac{{\left(\text{wt}.\;\text{of}\;CO_{2}\;\text{inside}\;\text{cell}\right)}_{P,T\;}}{{\left(\text{wt}.\;\text{of}\;\text{mPEG}-\text{alkyne}\right)}_{P,T;t=0}}$$

(2) $${\left(\text{wt}.\;\text{of}\;\text{mPEG}-\text{alkyne}\right)}_{P,T;t=0}=w_{f}\left(T,P\right)-w_{o}\left(T,P\right)$$

(3) $${\left(\text{wt}.\;\text{of}\;CO_{2}\text{inside}\;\text{cell}\right)}_{P,T}=\rho_{CO_{2}}\left(T,P\right)*\text{Volumen}\;\text{of}\;\text{the}\;\text{cell}$$

### 2.2 Solving Sanchez Lacombe equation of state for the mixing mPEG-alkyne/scCO_2_

Firstly, it was assumed that all the characteristic parameters and size parameter are known. The unknown parameter in the system of SL EOS are the mass fraction of CO_2_, density of polymer/CO_2_ mixture and ϕ_1_. It was resolved following the steps, as indicated the algorithm of the supplementary material:

Step 1: Set an initial value for parameter k_12_.

Step 2: w_f_ (T, P) and wo (T, P) are obtained with the help of high-pressure variable volume cell at given temperature and pressure condition. The mass fraction of CO_2_ was determined with Eq. (4).

(4) $${\left(\text{wt}.\;\text{of}\;CO_{2}\right)}_{P,T;t=0}=1-\left(w_{f}\left(T,P\right)-w_{o}\left(T,P\right)\right)$$

Step 3: With mass fraction of CO_2_, characteristic parameters, and k_12_(guess) it was determined the mixing parameters that appear in the manuscript file in Eqs. (8)–(14).

(5) $${\tilde{\rho }}^{2}+\tilde{P}+\tilde{T}\left[\ln\left(1-\tilde{\rho }\right)+\left(1-\frac{1}{r}\right)\tilde{\rho }\right]=0$$

(6) $$\tilde{P}=\frac{P}{P^{*}},\;\tilde{T}=\frac{T}{T^{*}},\tilde{\rho }=\frac{\rho }{\rho^{*}},\;\rho^{*}=\frac{\bar{M}}{v^{*}},r=\frac{v^{*}MP^{*}}{RT^{*}}$$

(7) $$\varphi_{1}^{o}=\frac{\varphi_{1}}{\varphi_{1}+\left(v_{1}^{*}\;/\;v_{2}^{*}\right)\varphi_{2}};\;\varphi_{1}^{o}+\varphi_{2}^{o}=1$$

(8) $$\varphi_{1}=\frac{\frac{m_{1}}{\rho_{1}^{*}}}{\frac{m_{1}}{\rho_{1}^{*}}+\frac{m_{2}}{\rho_{2}^{*}}};\;\varphi_{1}+\varphi_{2}=1$$

(9) $$P^{*}=\varphi_{1}P_{1}^{*}+\varphi_{2}P_{2}^{*}-\left(\frac{RT}{v^{*}}\right)\varphi_{1}\varphi_{2}X_{12}$$

(10) $$P_{12}^{*}=P_{1}^{*}+P_{2}^{*}-2{\left(P_{1}^{*}P_{2}^{*}\right)}^{\frac{1}{2}}\left(1-k_{12}\right)\frac{v^{*}}{RT}$$

(11) $$T^{*}=\frac{P^{*}v^{*}}{R}$$

(12) $$r=x_{1}r_{1}+x_{2}r_{2};\;x_{1}+x_{2}=1$$

(13) $$\frac{1}{\rho^{*}}=\frac{m_{1}}{\rho_{1}^{*}}+\frac{m_{2}}{\rho_{2}^{*}}$$

(14) $$\mu_{1}^{G}\left(T,P\right)=\;\mu_{1}^{P}\left(T,P,m_{1}\right)$$

(15) $$\frac{\mu_{1}^{P}}{RT}=ln\varphi_{1}+\left(1-\frac{r_{1}}{r_{2}}\right)\varphi_{2}+r_{1}\tilde{\rho }X_{12}\varphi_{2}^{2}\frac{v_{1}^{*}}{v^{*}}+r_{1}\left[\frac{-\tilde{\rho }+{\tilde{P}}_{1}\tilde{v}}{{\tilde{T}}_{1}}+\tilde{v}\left(\left(1-\tilde{\rho }\right)ln\left(1-\tilde{\rho }\right)+\frac{\tilde{\rho }ln\tilde{\rho }}{r_{1}}\right)\right]$$

(16) $$\frac{\mu_{1}^{G}}{RT}=r_{1}\left[\frac{-{\tilde{\rho }}_{1}+{\tilde{P}}_{1}{\tilde{v}}_{1}}{{\tilde{T}}_{1}}+{\tilde{v}}_{1}\left(\left(1-{\tilde{\rho }}_{1}\right)ln\left(1-{\tilde{\rho }}_{1}\right)+\frac{{\tilde{\rho }}_{1}\;ln\;{\tilde{\rho }}_{1}}{r_{1}}\right)\right]$$

Step 4: Knowing polymer/CO_2_ mixture reaches an equilibrium state at the considered temperature and pressure, the chemical potential of CO_2_ in both phases should be the same. Thus, it was compared Eqs. (13) and (17) and if the sum of square error between them is large we change the k_12_ value and go to step 1. When this difference was 0.0025 was considered acceptable, we stopped the iteration.

The characteristic parameters for CO_2_ can be determined from thermodynamic properties. However, there are numerous sources of characteristic parameters available in the literature. Several authors [4,^14^,15], suggest that the parameters of the pure components should be selected according to the temperatures and pressures at which the sorption isotherms are calculated. Therefore, pure component parameters determined at temperatures and pressures in the range of interest for sorption calculations should provide the best correlation of the sorption data. The supplementary material shows the characteristic parameters used to determine CO_2_ density at 308 K and 318 K. following Sanchez-Lacombe EOS and the sum of square error (SSE) between of density values from NIST database. Moreover, the supplementary material shows the critical properties for each characteristic parameter. The critical properties for the lattice fluid are a unique function of the r-mer size. The reduced critical properties Eqs. (17)–(19) are given for the characteristics parameters of SL EOS Eq. (5) [1]:

(17) $${\tilde{\rho }}_{c}=\frac{1\;}{1+r^{1/2}}$$

(18) $${\tilde{T}}_{c}=2r{{\tilde{\rho }}_{c}}^{2}$$

(19) $${\tilde{P}}_{c}={\tilde{T}}_{c}\left(\ln\left(1+\frac{1}{r^{2}}\right)+\left(\frac{1\;/\;2-r^{\frac{1}{2}}}{r}\right)\right)$$

### 2.3 Solving heuristic model

A heuristic model with experimental data has been correlated by Pasquali et al. [2] following the models proposed by Giddings et al. [16,17], where the solubility parameter can be expressed as a function of the CO_2_ solubility parameter and fitted by a second-degree equation:

(20) $$\log X=a\delta^{2}+b\delta +C$$

where X is the solute mole fraction, a and b are coefficients, C is a constant and δ is the solubility parameter of the CO_2_ at a given conditions. The solubility parameter of CO_2_ can be calculated by the equation:

(21) $$\delta =1.25Pc^{1/2}\frac{\rho r}{\rho r\left(liq\right)}$$

where P_c_, is the critical pressure and ρr, is the reduced density, which is the ratio of the apparent density of CO_2_ at given pressure and temperature to the critical density of CO_2_. The apparent density of CO_2_ has been calculated through the Bender equation and NIST. The solubility parameter of mPEG-alkyne was calculated with the Small method of group contributions. In order to calculate the solubility parameter, we used Eq. (20) with Small method.

(22) $$\delta_{i}=\frac{\rho_{p}\sum_{j}F_{j}\;}{M_{i}}$$

Where ρ_p_ is the polymer density and M_i_ is the molecular weight of polymers, δ_i_ can be evaluated for repeating group by using group contribution calculations for the molar volume and the cohesive energy density or molar attraction constant. These parameters were calculated as described in previous sections.

### 2.4 Henry law

The polymer/scCO_2_ mixture undergoes throughout different states, being the first one a liquid or rubbery state, where the absorption of CO_2_ into the polymer generally follows Henry's law as shown in Eq. (23). In this law the mass fraction of CO_2_ absorbed (w_CO_2__) is proportional to the partial pressure of the scCO_2_, P CO_2_. It is easy to find in literature the Henry's Constant value (k_H_) for many polymers, being in this case the k_H_ value for PEG 0.0198 wt.fraction/MPa [10]. The algorithm for using Henry's Law is attached in the supplementary material.

(23) $$w_{CO_{2}}=k_{H}P_{CO_{2}}$$

### 2.5 Dual-mode sorption

The dual mode sorption model, Eq. (24), is a combination of Henry's law in the equilibrium zone and Langmuir type sorption in the non-equilibrium zone. Henry's constant has the same physical meaning for glassy polymers than for rubbery polymers and liquids, whereas the Langmuir-type term account for gas sorption into interstitial spaces and microvoids, which are consequences of local heterogeneities and are intimately related to the slow relaxation processes associated with the glassy state of the polymers.

(24) $$S=\;k_{H}P+\frac{c{}_{H}^{\prime }bP}{1+bP}$$

Where S is the sorption of CO_2_ in the polymer, k_H_ is analogous to Henry's law constant, P is the pressure, $c{{}^{\prime }}_{H}$ is the saturation of the cavities and b represents the affinity between the solute molecules and the Langmuir sites present in the polymeric matrix. For this work the values of $c{{}^{\prime }}_{H}$ and b were determined with an Excel spreadsheet tool (Solver system). This system used the nonlinear programming algorithm generalized reduced gradient (GRG). The algorithm for using Dual Mode is attached in the supplementary material.

## Declaration of Competing Interest

The authors declare that they have no know competing financial interests or personal relations-ships that could have appeared to influence the work reported in this paper.

## Appendix Supplementary materials

Image, application 1
